# Newly Developed Di-Block Copolymer-Based Cell Membrane Stabilizers Protect Mouse Coronary Artery Endothelial Cells against Hypoxia/Reoxygenation Injury

**DOI:** 10.3390/cells12101394

**Published:** 2023-05-15

**Authors:** Zhu Li, Mukesh K. Gupta, Matthew B. Barajas, Takuro Oyama, Craig L. Duvall, Matthias L. Riess

**Affiliations:** 1 Department of Anesthesiology, Vanderbilt University Medical Center, Nashville, TN 37232, USA; zhu.li@vumc.org (Z.L.); matthew.b.barajas@vumc.org (M.B.B.); takuro.oyama@vumc.org (T.O.); 2 Department of Biomedical Engineering, Vanderbilt University, Nashville, TN 37232, USA; mukesh.k.gupta@vanderbilt.edu (M.K.G.);; 3 Anesthesiology, TVHS VA Medical Center, Nashville, TN 37212, USA; 4 Department of Pharmacology, Vanderbilt University, Nashville, TN 37232, USA

**Keywords:** apoptosis, heart, ischemia reperfusion injury, LDH, myocardial, murine, P188, poloxamer, tert-butyl, tri-block

## Abstract

Reperfusion after ischemia causes additional cellular damage, known as reperfusion injury, for which there is still no effective remedy. Poloxamer (P)188, a tri-block copolymer-based cell membrane stabilizer (CCMS), has been shown to provide protection against hypoxia/reoxygenation (HR) injury in various models by reducing membrane leakage and apoptosis and improving mitochondrial function. Interestingly, substituting one of its hydrophilic poly-ethylene oxide (PEO) blocks with a (*t*)ert-butyl terminus added to the hydrophobic poly-propylene oxide (PPO) block yields a di-block compound (PEO-PPO*t*) that interacts better with the cell membrane lipid bi-layer and exhibits greater cellular protection than the gold standard tri-block P188 (PEO_75_-PPO_30_-PEO_75_). For this study, we custom-made three different new di-blocks (PEO_113_-PPO_10_*t*, PEO_226_-PPO_18_*t* and PEO_113_-PPO_20_*t*) to systemically examine the effects of the length of each polymer block on cellular protection in comparison to P188. Cellular protection was assessed by cell viability, lactate dehydrogenase release, and uptake of FM1-43 in mouse artery endothelial cells (ECs) following HR injury. We found that di-block CCMS were able to provide the same or better EC protection than P188. Our study provides the first direct evidence that custom-made di-block CCMS can be superior to P188 in improving EC membrane protection, raising their potential in treating cardiac reperfusion injury.

## 1. Introduction

Timely reperfusion through restoring blood flow is the only way to salvage cardiac tissue after an ischemic event. However, reperfusion itself can cause significant additional cellular injury as a result of sudden reintroduction of oxygen to previously hypoxic tissue. This is known as myocardial reperfusion injury, a condition for which there is no effective remedy. Reperfusion can cause endothelial cell (EC) dysfunction [1,2], cell membrane depolarization [3,4], aggravation of intracellular calcium overload [5,6], and apoptotic cell death [7,8,9,10].

In vivo, ischemia and reperfusion (IR) damage different types of cells in different ways. Cardiomyocytes (CMs) and ECs are the two most abundant cardiac cell types. In the heart, the pathological response to IR mostly involves inflammation and oxidative stress of cardiomyocytes, but IR also damages surrounding cells, especially ECs. EC dysfunction aggravates CM injury and consequently results in injury expansion, possibly leading to cell death. On the other side, ECs can also provide cytoprotection for CMs during IR, highlighting one of the crucial roles of EC-CM crosstalk [11].

Our previous study [12] used a mouse isolated CM model to investigate the role of reperfusion alone on cell membrane disruption by reoxygenating cells following hypoxia (HR as simulated IR). It demonstrated that, after 5 h hypoxia followed by 2 h reoxygenation, cell viability decreased and lactate dehydrogenase (LDH) release increased significantly compared to either normoxia or hypoxia alone, indicating cell membrane function was strongly affected as a result. The study also employed Poloxamer (P)188, a copolymer-based cell membrane stabilizer (CCMS) given at the clinically and translationally relevant time of reoxygenation following hypoxia, to demonstrate its protective effects on cell morphology, LDH release, calcium influx, and insertion of the fluorescent membrane probe FM1-43. These findings suggest that CCMS such as P188 can be used effectively in reducing HR-caused damage to cells and, thus, might represent an attractive solution to reperfusion injury.

P188 is a tri-block CCMS consisting of a core lipophilic poly-propylene oxide (PPO) block of 30 units, flanked by two hydrophilic poly-ethylene oxide (PEO) blocks of 75 units to form a PEO_75_-PPO_30_-PEO_75_ structure of 8.4 kDa. As such, P188 has both hydrophilic and lipophilic components. By varying the block lengths or numbers of the PPO and PEO units, different forms of CCMS can be designed to achieve different chemical properties. In particular, the hydrophilic PEO alone of comparable molecular weight known as polyethylene glycol (PEG) does not appear to protect cell membrane function when used in parallel experiments as osmotic control [12,13,14]. Supporting this notion, a recent study of structure modifications of P188 found that certain di-block compounds (PEO-PPO) can provide stronger protection than P188 against cellular damage in muscle myoblasts undergoing hypotonic shock and that modification of the lipophilic PPO terminal—such as adding a (*t*)ert-butyl end—can increase this protection even more [15].

**Figure 1:**
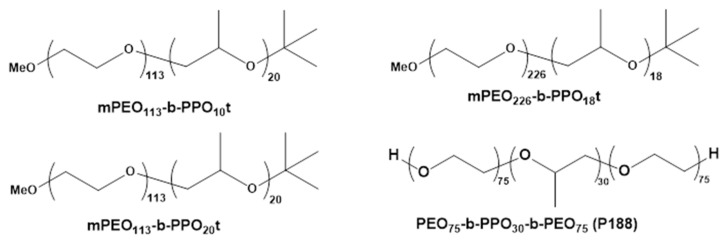
ChemDraw schematic for the three di-block CCMS and P188.

In this study, we tested the hypothesis that newly developed di-block CCMS can provide profound protection against injury by prolonged HR in mouse coronary artery ECs when given as a postconditioning agent upon reoxygenation. To this end, we custom-made three different di-block CCMS: PEO_113_-PPO_10_*t*, PEO_226_-PPO_18_*t,* and PEO_113_-PPO_20_*t* (Figure 1). This enabled systematic examination of the effects of polymer chemical alterations on cell membrane protection by evaluating cell viability, LDH release, and facilitation of FM1-43 insertion. These new polymers were compared to the tri-block CCMS P188 (Figure 1), and the overall goal was to identify new polymer compositions that will enable better clinical outcomes in the future.

## 2. Materials and Methods

P188 (Kolliphor P 188; catalog number K4894) was purchased from Sigma (St. Louis, MO, USA).

### 2.1. Di-Block Copolymer Synthesis

**Figure A1:**
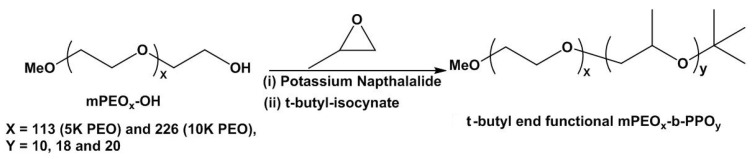
Synthesis of *t*-butyl end functional di-block copolymers of mPEO_x_-b-PPO_y_.

**Figure A2:**
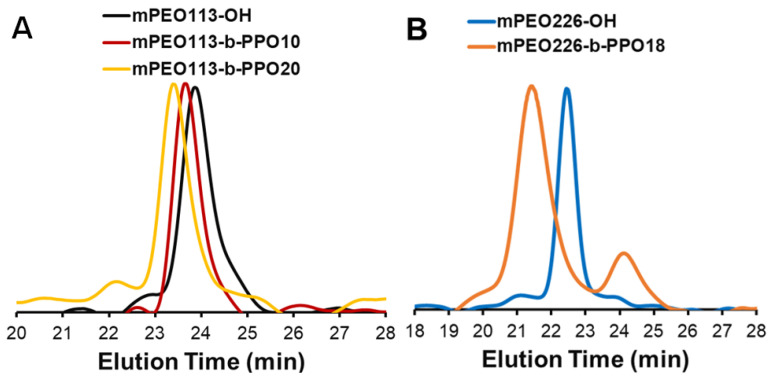
GPC chromatogram of tertiary butyl end functional di-block copolymers. (**A**) 5K PEO-based mPEO_113_-b-PPO_10_*t* and mPEO_113_-b-PPO_20_*t* and (**B**) 10 kDa PEO-based mPEO_226_-b-PPO_18_*t*. A clear left-hand side shift in GPC chromatogram for di-block copolymers relative to parent PEO polymer indicated successful di-block copolymers.

**Figure A3:**
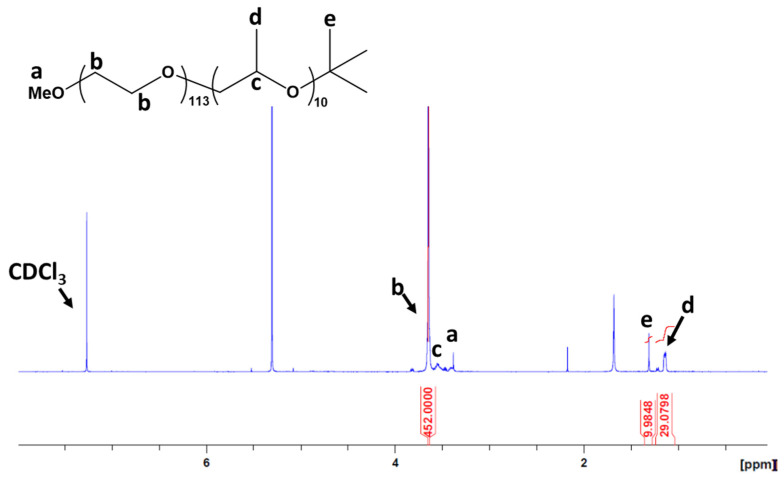
^1^H NMR spectra of tertiary butyl end functional di-block copolymer of mPEO_113_-b-PPO_10_*t*. The presence of characteristic peaks indicated the formation of a successful di-block copolymer. Letters a to e in the spectra correspond to the position in the molecule.

**Figure A4:**
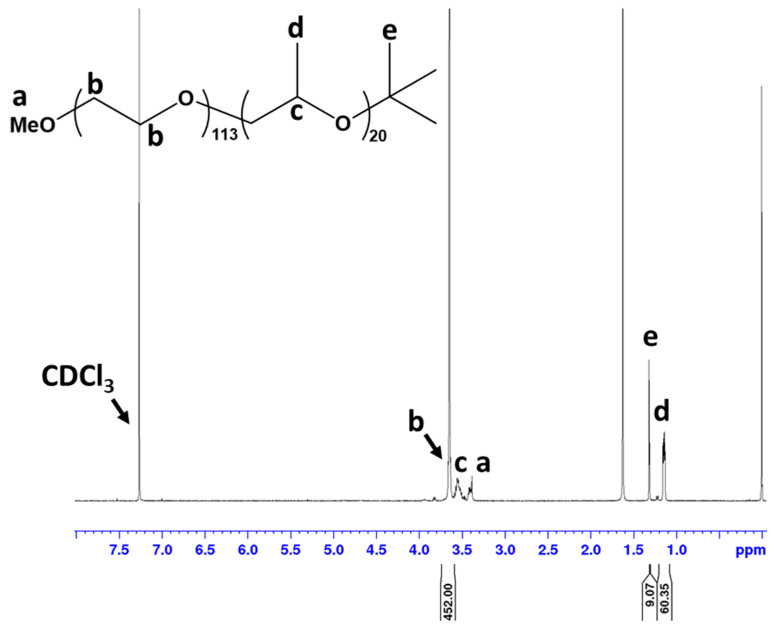
^1^H NMR spectra of tertiary butyl end functional di-block copolymer of mPEO_113_-b-PPO_20_*t*. Letters a to e in the spectra correspond to the position in the molecule.

**Figure A5:**
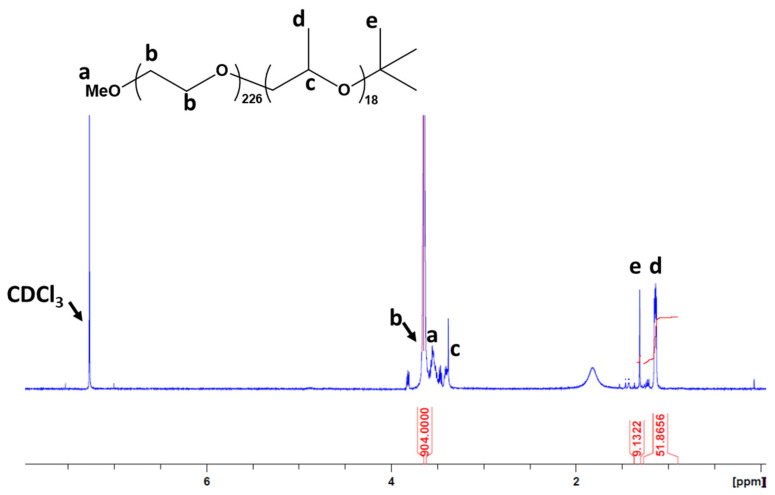
^1^H NMR spectra of tertiary butyl end functional di-block copolymer of mPEO_226_-b-PPO_18_*t*. Letters a to e in the spectra correspond to the position in the molecule.

The three di-blocks were custom-made by authors MKG and CLD. To prepare the three *t*-butyl end functional di-blocks, PEO_113_-b-PPO_10_*t*, PEO_226_-b-PPO_18_*t*, and PEO_113_-b-PPO_20_*t*, methoxy poly(ethylene glycol) (mPEG) of a molecular weight of either 5 or 10 kDa was used for ring-opening polymerization of propylene oxide (PO) using potassium naphthalide as an initiator. Resulting polymers were end-capped using *t*-butyl isocyanate (Figure 1 and Figure A1). The formation of di-block copolymer (mPEG-b-PPO*t*) was characterized by gel permeation chromatography (GPC) and ^1^H NMR (Figure A2, Figure A3, Figure A4 and Figure A5). Their molecular weights are 5552, 10,988, and 6132 Da, respectively. The di-blocks have significantly shorter PPO units than P188.

### 2.2. Cell Culture and In Vitro HR Injury

C57BL/6 mouse primary coronary artery ECs (Catalog No. C57-6093) were purchased from Cell Biologics (Chicago, IL, USA). They were isolated from coronary arteries of pathogen-free laboratory mice. Kept in growth media containing 10% fetal bovine serum (FBS) and antibiotics, the cells were cultured in a cell incubator with a standard humidified culture environment of 21% O_2_, 5% CO_2_, 74% N_2_ at 37 °C.

**Figure 2:**
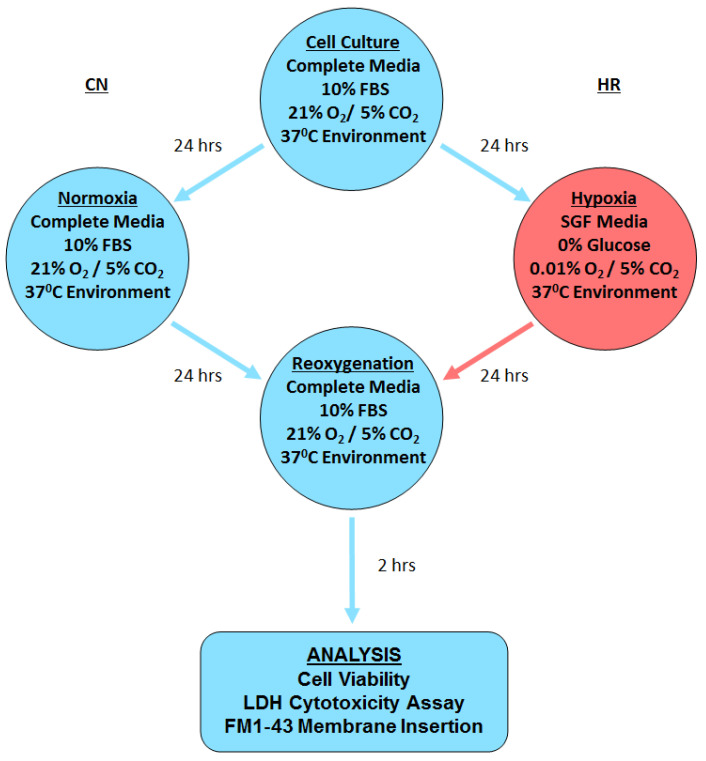
Flow chart of endothelial injury model. CN: control/normoxia; HR: hypoxia/reoxygenation; FBS: Fetal Bovine Serum; SGF: serum- and glucose-free; LDH: lactate dehydrogenase.

Figure 2 shows the flow chart of our hypoxic injury model. Cells were plated in regular media at a density of 10,000 cells per well into 96-well plates. Multiple replicates for each control and treatment group were plated in each experiment. After about 24 h, when the cells reached confluency, the plates of cells for each experiment were randomized to control normoxia (CN) or HR groups. To simulate IR by starvation of O_2_ and nutrients, the cells were placed into serum- and glucose-free (SGF) media in a humidified Billups-Rosenthal plexiglass hypoxia chamber (Stemcell Technologies; Vancouver, BC, Canada), flushed with a hypoxic gas mixture (0.01% O_2_; 5% CO_2_, 95% N_2_) and placed in the cell incubator at 37 °C for 24 h. Note that this insult is stronger than previously used in CMs (see below) [12]. CN cells were kept under normal conditions; regular serum- and glucose-containing cell media was refreshed at the same time points as the corresponding HR cells. The 2-h reoxygenation period started when the cells were removed from the hypoxic chamber. The media was refreshed with regular media, and the experimental plates returned to the normal culture environment (21% O_2_, 5% CO_2_, 74% N_2_, 37 °C).

The rationale for 24 h of hypoxia and 2 h of reoxygenation is as follows: We have previously found that 5 h hypoxia followed by 2 h reoxgenation caused significant cell death, increased LDH release and FM1-43 dye membrane insertion in CMs. This damage was attenuated by P188 treatment. However, in the present EC model, the magnitudes of HR damage were not consistently as high as in CMs after 5 h hypoxia/2 h reoxygenation; in particular, LDH release did not increase 2- to 3-fold as desired and previously observed in prior models. Therefore, after several preliminary dose-response experiments, we prolonged the hypoxia duration to 24 h.

### 2.3. Cell Viability Assay

To assess cell viability, the CyQUANT Direct Cell Proliferation Assay Kit (Catalog number A13261) from Invitrogen (Eugene, OR, USA) was used. It measures fluorescent intensity of the stain binding to the DNA in alive cells. By following the assay protocol, at the end of the 2-h reoxygenation period, cells were washed with phosphate buffered saline (PBS) before adding 100 µL of fresh regular media with staining dye appropriately diluted. After 60 min incubation at 37 °C, the plates were read at an excitation (ex) of 480 nm and emission (em) of 535 nm in a plate reader (Synergy H1; BioTek Instruments Inc, Winooski, VT, USA).

### 2.4. Assessment of Cell Membrane Injury and Repair

Cell membrane injury and repair were assessed by two different methods in all experiments.

#### 2.4.1. LDH Cytotoxicity Assay

The first method was the measurement of the intracellular enzyme LDH released from damaged cells into the culture media using the LDH Cytotoxicity Assay Kit (Pierce Biotechnology, Rockford, IL, USA). LDH leakage is widely used as an indicator of reduced cell membrane integrity and consequent cytotoxicity.

Following the assay protocol, at the end of the 2-h reoxygenation period, 50 µL of media from each well to be used was transferred to a corresponding well of a new 96-well plate and mixed with 50 µL of prepared LDH reaction mixture. After 30 min incubation at room temperature and protected from light, the reactions were terminated by the addition of 50 µL of stop solution, which halts the reduction of the tetrazolium salt to the formazan product. Absorbance of each well was measured at 490 nm using the plate reader.

#### 2.4.2. FM1-43 Membrane Insertion

The membrane impermeant styryl dye FM1-43 (Molecular Probes, Inc, Eugene, OR, USA) was utilized as a second, independent measure of cell membrane damage as well as repair. This is a validated marker utilized in a multitude of cell lines [12,16,17]. FM1-43 remains extracellular unless damage to the membrane allows it to become incorporated into the lipid bilayer of the cell membrane, where it specifically fluoresces. Following the method of Yasuda et al. [18], 2.5 µM FM1-43 was added to the media at the start of the reoxygenation period. At the end of the experiment, the cells were washed with 1 × PBS to remove any remaining extracellular, non-incorporated dye. Then, 100 µL of 1 × PBS was added to the wells, and the fluorescence at ex = 488 nm and em = 568 nm was read from the bottom on a plate reader.

### 2.5. Treatment with P188 and Di-Block CCMS

All CCMS were administered at the start of reoxygenation and tested at four different concentrations (0, 30, 100, and 300 µM). This range was chosen based on other studies that have investigated the cellular protective potential of P188: in mouse muscle myoblast cultures (~80% confluent) exposed to hypo-osmotic stress and isotonic recovery, 14 µM P188 was protective; 150 µM P188 fully restored dystrophic mouse myocyte stretch compliance; and 0.5 mM P188 repaired cultured astrocytes that had been exposed to traumatic-brain-injury-induced microcavitation. All CCMS were dissolved in regular cell media to achieve the desired concentrations (30 to 300 µM). At these concentrations, it is ≥96% soluble, does not affect the media’s pH, and exists as a dynamic solution of single molecules as well as grouped micelles [19]. Di-blocks work in a similar fashion to P188, sealing the holes in the cell membrane. Therefore, dose-equivalency is expected.

### 2.6. Statistics

All experiments were repeated at least 4 times. All data are normalized to %CN in the absence of CCMS (0 µM) and expressed as mean +/- standard error of the mean (SEM). Statistical differences between groups were analyzed by analysis of variance followed by Student-Newman-Keuls post hoc comparisons. A *p* value of <0.05 (two-tailed) was considered to indicate statistically significant differences compared to * CN or # 0 µM CCMS, † 100 µM CCMS or § 300 µM CCMS under HR conditions. Statistical analyses were performed using Sigma Stat 3.5 (Systat Software, San Jose, CA, USA).

## 3. Results

### 3.1. HR Injury in ECs

**Figure 3:**
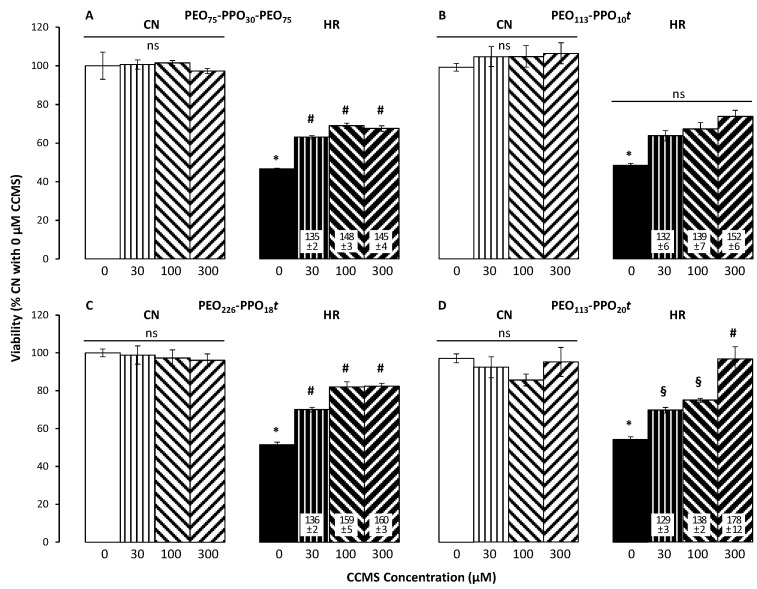
Effect of different CCMS concentrations (0 to 300 µM) on cell viability under control/normoxic conditions (CN, left brighter group in each panel) vs. after hypoxia/reoxygenation (HR, right darker group in each panel) as measured by the CyQUANT Direct Cell Proliferation Assay Kit (n = 5–8 per group). Panel (**A**): P188; panels (**B**–**D**): different di-blocks (PEO_113_-PPO_10_*t*, PEO_226_-PPO_18_*t,* and PEO_113_-PPO_20_*t*), respectively. Data are mean +/− standard error of the mean and normalized to %CN without CCMS (white bars). Numbers in white boxes are %HR without CCMS (black bars). Statistical symbols: *p* < 0.05 * vs. 0 µM CCMS under CN conditions, # vs. 0 µM CCMS under HR conditions, § vs. 300 µM CCMS under HR conditions. There was no statistically significant difference (ns) among any concentrations under CN conditions for any CCMS.

**Figure 4:**
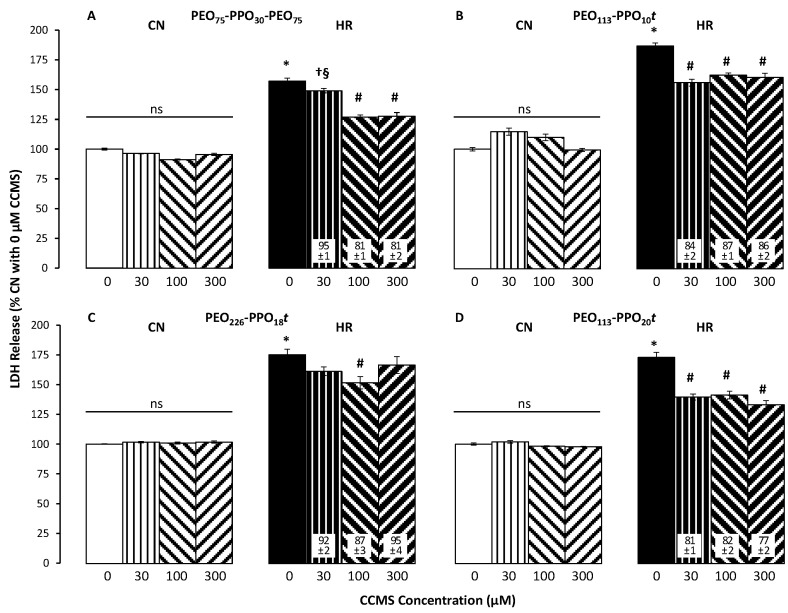
Effect of different CCMS concentrations (0 to 300 µM) on membrane injury under control/normoxic conditions (CN, left brighter group in each panel) vs. after hypoxia/reoxygenation (HR, right darker group in each panel) as measured by Lactate Dehydrogenase (LDH) Cytotoxicity Assay Kit (n = 5–8 per group). Panel (**A**): P188; panels (**B**–**D**): different di-blocks (PEO_113_-PPO_10_*t*, PEO_226_-PPO_18_*t,* and PEO_113_-PPO_20_*t*, respectively). Data are mean +/− standard error of the mean and normalized to %CN without CCMS (white bars). Numbers in white boxes are %HR without CCMS (black bars). Statistical symbols: *p* < 0.05 * vs. 0 µM CCMS under CN conditions, # vs. 0 µM CCMS under HR conditions, † vs. 100 µM CCMS under HR conditions, § vs. 300 µM CCMS under HR conditions. There was no statistically significant difference (ns) among any concentrations under CN conditions for any CCMS.

**Figure 5:**
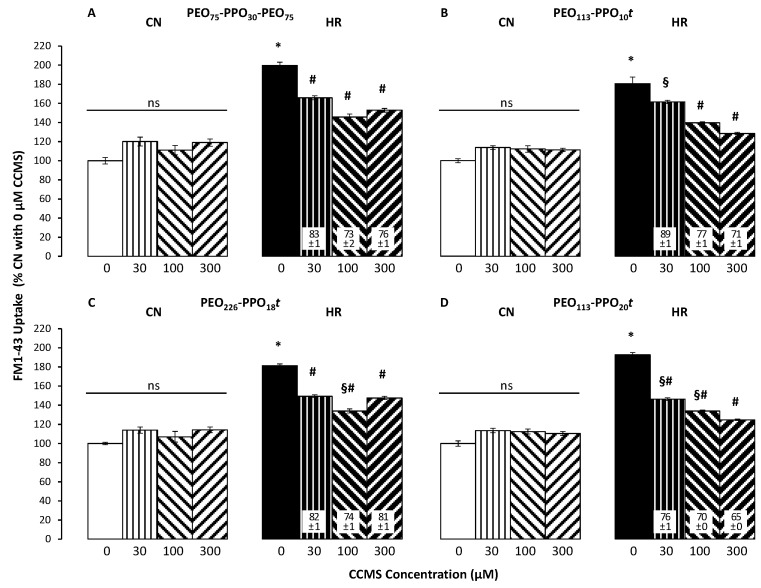
Effect of different CCMS concentrations (0 to 300 µM) on membrane injury under control/normoxic conditions (CN, left brighter group in each panel) vs. after hypoxia/reoxygenation (HR, right darker group in each panel) as measured by uptake of the membrane impermeant styryl dye FM1-43 (n = 4 per group). Panel (**A**): P188; panels (**B**–**D**): different di-blocks (PEO_113_-PPO_10_*t*, PEO_226_-PPO_18_*t,* and PEO_113_-PPO_20_*t*, respectively). Data are mean +/− standard error of the mean and normalized to %CN without CCMS (white bars). Numbers in white boxes are %HR without CCMS (black bars). Statistical symbols: *p* < 0.05 * vs. 0 µM CCMS under CN conditions, # vs. 0 µM CCMS under HR conditions, § vs. 300 µM CCMS under HR conditions. There was no statistically significant difference (ns) among any concentrations under CN conditions for any CCMS.

Two hours of reoxygenation following 24 h of hypoxia resulted in significant EC death compared to CN. In the absence of CCMS, HR reduced cell number/viability to about half (Figure 3). Corresponding results were seen in studies assessing cell membrane damage: HR significantly increased LDH release by 60 to 90% over CN levels (Figure 4). FM1-43 intensity in HR cells was 80 to 100% higher than in CN cells (Figure 5).

### 3.2. Effects of P188 and Di-Block CCMS on HR Injury

CCMS were added to fresh medium at the beginning of reoxygenation for 2 h to treat the cells that had been maintained with or without 24 h of hypoxia.

#### 3.2.1. Cell Viability

As seen in Figure 3A, P188 treatment during reoxygenation significantly improved the cell viability of HR cells at all tested concentrations, from as low as 30 µM to as high as 300 µM compared to 0 µM. Even with the highest concentration of 300 µM, though, cell viability did not reach CN levels. Under CN conditions, P188 did not alter cell viability.

The di-block with the shortest PPO length, PEO_113_-PPO_10_*t* (Figure 3B), showed a similar pattern of cell protection as P188 but did not reach statistical significance compared to vehicle (*p* = 0.09). However, protection was improved when PEO_226_-PPO_18_*t* (Figure 3C) and PEO_113_-PPO_20_*t* (Figure 3D) were given, with the latter maintaining a higher level of viability than the former by reaching CN levels at 300 µM. Under CN conditions, neither of the di-blocks altered cell viability significantly.

#### 3.2.2. LDH Release

Under CN conditions, there was no statistically significant difference among any concentrations for any CCMS. P188 at 100 µM and 300 µM reduced LDH release under HR conditions significantly (Figure 4A). Similar to cell viability, PEO_113_-PPO_20_*t* (Figure 4D) resulted in a stronger dose-dependent decrease in LDH release under HR conditions than PEO_113_-PPO_10_*t* (Figure 4B) or PEO_226_-PPO_18_*t* (Figure 4C).

#### 3.2.3. FM1-43 Membrane Insertion

Figure 5 shows that there was no statistically significant difference in FM1-43 fluorescent intensity among any concentrations for any CCMS under CN conditions. However, in HR cells, P188 as well as all three di-blocks dose-dependently reduced FM1-43 fluorescence, with PEO_113_-PPO_20_*t* again showing the strongest effect by almost reaching CN levels at 300 µM.

## 4. Discussion

### 4.1. Establishing the HR Model in ECs

Establishing pertinent in vitro cell culture models for studying simulated IR injury and its mitigation has become an important first step to understand and develop solutions to IR injury after an ischemic event. Whether different cell types and tissues might respond differently to IR injury remains unclear, and so does whether CCMS, such as P188, differentially affect varied cell types. In a previous study [12], we used mouse isolated CMs to show that in vitro simulation of myocardial IR allowed measurements of cell membrane-related activities, such as cell viability, LDH released through cell membranes, and the fluorescent dye FM1-43 inserted into membranes. In this study, we used a mouse coronary artery EC culture undergoing HR in vitro to simulate and evaluate in vivo IR injury in a different but highly relevant cardiac cell type, ECs.

Our preliminary data indicated that isolated ECs are more resistant to HR injury: 5 h hypoxia followed by 2 h reoxygenation did not produce as much damage in ECs as in isolated CMs, requiring extension of simulated IR injury to 24 h. Under these conditions, viability was largely decreased, and LDH release and FM1-43 intensity largely increased. Importantly, despite the resultant severe level of damage, we were able to attenuate these injuries using CCMS. Our insult mimics severe but salvageable IR injury with potential rescue by pharmacologic postconditioning. Therefore, the current EC HR model is useful to evaluate the cardioprotective effect of CCMS di-blocks in direct comparison to P188.

### 4.2. Protection by P188

P188 is a tri-block CCMS. Its amphiphilic structure with a hydrophobic PPO block in the center and two hydrophilic PEO blocks on each side provides a molecule with diverse properties and potential for biomedical and pharmaceutical applications [20]. It has been used for many years in studies of IR injury in animals [18,21,22,23,24,25] as well as clinically in patients [26,27]. Its mechanism is thought to be reduction in membrane leakage [13,26,28], and apoptosis [29] and improvement in mitochondrial function in vivo [24] but not necessarily in vitro [30,31]. P188 has several additional biological applications such as a drug delivery facilitator, drug penetration enhancer, etc. [27,32]. All these properties are directly linked to its varying affinity to amphiphilic phospholipid membranes.

Copolymer-based CCMS have been demonstrated to directly interact with cell membranes by sealing permeable pores caused by oxidative stress and, thus, stabilize the membranes [13,28,33]. The triblock copolymer P188 is the most-studied CCMS to date. P188 has been shown [13,34] to insert into the lipid monolayer of membranes when surface pressure is low, indicating that P188 only inserts into damaged cell membranes with reduced lipid density and, thus, a lower surface pressure. Once the membrane surface pressure returns to normal, P188 is released and, therefore, becomes available to insert into other damaged membranes. P188 does not seem to cross the lipid bilayer membrane at low concentrations, thus not entering the cytoplasm. These attributes make it a suitable compound to determine if stabilization of cell membranes mitigates the damage caused by HR.

Various HR injury models in vivo and in vitro [35] have suggested that P188 can attenuate and even prevent or reverse IR injury. Instead of before or during the hypoxic period, we administered P188 at the beginning of the 2-h reoxygenation period, which is clinically highly relevant as—in contrast to most ischemic events—the onset of reperfusion is usually known. During reperfusion, the cells suffer the most severe damage. This includes the lowest cell viability and the worst membrane leakage, indicated by LDH release and FM1-43 incorporation, which makes reperfusion the critical turning point to intervene pharmacologically. Similar to a previous study in mouse brain microvascular ECs [36], our data indicate that P188 treatment upon reperfusion produced a dose-dependent attenuation of membrane leakage and cell death in mouse coronary artery ECs.

### 4.3. Protection by Di-Block CCMS

Interestingly, we observed that PEO_113_-PPO_20_*t,* with its longer lipophilic PPO and strongly lipophilic tert-butyl terminal, consistently exhibited a stronger attenuation of LDH release, FM1-43 uptake and improvement than the other two tested di-blocks.

These findings in our EC model allow several conclusions: First, the tri-block configuration of P188 (PEO_75_-PPO_30_-PEO_75_) is not absolutely necessary to provide cell membrane protection and, thus, cellular protection against HR injury. In fact, flanking the lipophilic PPO block with hydrophilic PEO blocks on either side prevents chemical modifications and, thus, improvements in the PPO block to anchor better in the cell membrane; only the change to a di-block CCMS allows this important modification [37]. Second, although the addition of a tert-butyl terminal renders the already lipophilic PPO block even more lipophilic to allow for better anchoring in the cell membrane, the PPO block itself still has to be long enough for optimal anchoring (PEO_113_-PPO_20_*t* is more potent than PEO_113_-PPO_10_*t* with its shorter PPO). Third, the length of the hydrophilic PEO block also appears to play a noteworthy role (PEO_226_-PPO_18_*t* is consistently not as protective as PEO_113_-PPO_20_*t* with their different PEO lengths being the main difference between them). This suggests that there is an optimal PEO length beyond which protection decreases again (PEO_113_-PPO_20_*t* being more potent than PEO_226_-PPO_18_*t*) [15].

### 4.4. Limitations and Outlook

Our in vitro approach lacks the physiological complexity of, and thus cannot replace, future in vivo experiments; at the same time, it does allow the separate study of different cell types that in vivo experiments do not. We had to titrate the extent of simulated IR, i.e., HR, to the given resistance of our cultured ECs against injury; in order to eventually study the effect of P188 and di-block CCMS in co-culture [17], we will have to use the same hypoxia durations for both ECs and CMs, though. The co-culture model will also allow us to study mechanisms of interaction (cross-talk) between ECs and CMs [38]. Longer hypoxic times may also inform us on the potential protection by CCMS when β-oxidation and glycolysis become more important [39]. Although we studied a range of different di- and tri-block CCMS concentrations, we only applied them at the time point of reoxygenation. Whether a similar, better, or lesser degree of protection can be observed with earlier vs. later administration will need to be elucidated as well; later administration, however, did not seem to work in an in vivo model of cardiac IR [24]. Finally, we only studied three different outcome parameters and three different di-block CCMS by systematically altering their PEO and PPO lengths, respectively; while a more comprehensive exploration was beyond the scope of this study, it is conceivable that assessment of further parameters such as oxidative stress and altered mRNA or protein expression as well as fine-tuning of PEO/PPO lengths might lead to more knowledge and even better CCMS compounds, respectively.

## 5. Conclusions

In summary, our data, for the first time, demonstrate that not just CMs [12] but also coronary artery ECs can be protected against simulated cardiac IR injury by postconditioning with CCMS. Our data also show, for the first time, that newly designed di-block CCMS can be as effective as, or even superior to, the current gold standard, the tri-block CCMS P188 in protecting coronary artery ECs against HR injury. Remarkably, the degree of EC protection by di-block CCMS strongly depends on their chemical composition, i.e., mostly the length of the hydrophobic PPO block to optimally anchor in the cell membrane as well as the length of their hydrophilic PEO block. Thus, di-block CCMS have the translational potential to become effective new compounds in mitigating cardiac reperfusion injury in future clinical practice.

## Data Availability

Original data are available to the interested reader upon reasonable request and in accordance with federal guidelines set forth by the funding agencies.

## Appendix A

Synthesis and characterization of tertiary butyl end functional di-block copolymers of mPEO_x_-b-PPO_y_:

The schematic for synthesizing di-block copolymers is shown in Figure A1.

To prepare di-block copolymers, methoxy poly(ethylene glycol) (mPEG-OH) was used for ring-opening polymerization of propylene oxide (PO) using potassium naphthalide as an initiator followed by termination of polymerization using *t*-butyl isocyanate. The representative procedure for mPEO_113_-b-PPO_10_ is as follows: In a flame-dried septum-fitted Schlenk tube, azeotropically dried mPEO_113_-OH (0.2 mmol, 1 g) was dissolved in anhydrous tetrahydrofuran (5 mL) under high-purity nitrogen. To this solution, a freshly prepared 1 M solution of potassium naphthalide (0.24 mmol, 0.040 mL) was transferred and stirred for 30 min. Next, dried propylene oxide (4 mmol, 0.232 g, 0.270 mL) was added and submerged in an oil bath at 45 °C for 24 h. The polymerization was terminated by adding 10% acetic acid in methanol and precipitated into cold diethyl ether. The formation of di-block was characterized by ^1^H NMR and GPC. Figure A2A shows GPC chromatograms for mPEO_113_-OH (5 kDa PEO), mPEO_113_-b-PPO_10_, and mPEO_113_-b-PPO_20_. The relative shift for mPEO-b-PPO di-block copolymers compared to parent mPEO indicated successful di-block formation. The same trend was observed for GPC chromatograms for mPEO_226_-OH (10 kDa PEO) and mPEO_226_-b-PPO_18_ (Figure A2B). The prepared di-block copolymers reacted with ten molar excess of *t*-butyl isocyanate in the presence of catalytic dibutyltin dilaurate for 24 h to yield *t*-butyl end functional di-block copolymers. The respective ^1^H NMR spectra for mPEO_113_-b-PPO_10_*t* (Figure A3), mPEO_113_-b-PPO_20_*t* (Figure A4), and mPEO_226_-b-PPO_18_*t* (Figure A5) show peaks for both mPEO-OH (3.65 ppm) and PPO (methyl peak at 1.1 ppm) blocks. The characteristic peak at 1.3 ppm indicated successful end functionalization of di-block copolymers with the *t*-butyl group. The collective data from ^1^H NMR and GPC demonstrated the successful synthesis of respective di-block copolymers.
